# Supplementary material for: Omnidirectional Triboelectric Nanogenerator for Wide-Speed-Range Wind Energy Harvesting
Source: Nanomaterials (Basel). 2022 Nov 17;12(22):4046. doi: 10.3390/nano12224046 (PMC9698673; doi:10.3390/nano12224046)
Supplement: Supplementary file 1 [file nanomaterials-12-04046-s001.zip › nanomaterials-2007343-supplementary.pdf]

Supplementary Materials

Omnidirectional Triboelectric Nanogenerator for Wide-Speed-Range Wind Energy Harvesting

Qiman Wang [1], Wenhao Li [2], Kun Wang [2], Yitao Liao [2], Junjie Zheng [2], Xiongtu Zhou [1,2,3], Jianpu Lin [1,*], Yongai Zhang [1,2,3,*] and Chaoxing Wu [1,2,3,*]

1 College of Advanced Manufacturing, Fuzhou University, Quanzhou 362251, China
2 College of Physics and Information Engineering, Fuzhou University, Fuzhou 350108, China
3 Fujian Science and Technology Innovation Laboratory for Optoelectronic Information of China, Fuzhou 350108, China
* Correspondence: ljp@fzu.edu.cn (J.L.); yongaizhang@fzu.edu.cn (Y.Z.); chaoxing_wu@fzu.edu.cn (C.W.)

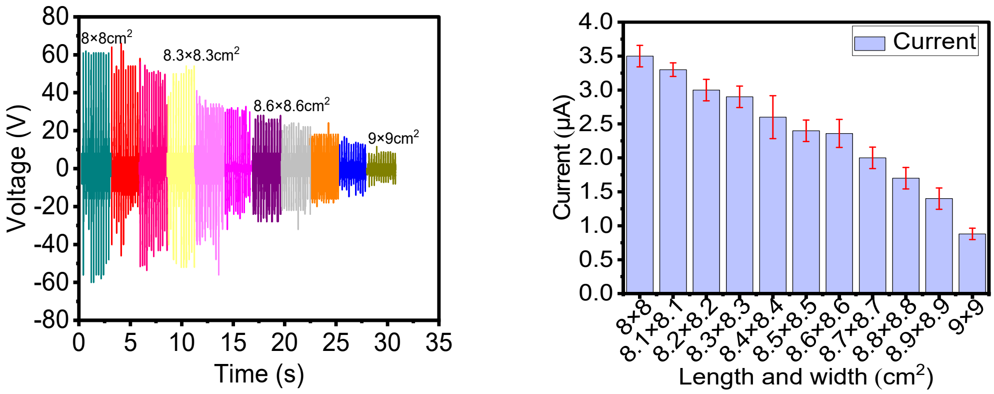

Figure S1 V_{OC} and I_{SC} of the CS-TENG with different inner box areas.
